# Compositional design and Taguchi optimization of hardness properties in silicone-based ocular lenses

**DOI:** 10.1007/s40204-017-0065-y

**Published:** 2017-05-15

**Authors:** Mohammad Hanifeh, Mojgan Zandi, Parvin Shokrollahi, Mohammad Atai, Ebrahim Ghafarzadeh, Fahimeh Askari

**Affiliations:** 1 Biomaterials Department, Iran Polymer and Petrochemical Institute, Pazhoohesh Blvd., Tehran-Karaj Hwy, Tehran, 1497713115 Iran; 2 Science Department, Iran Polymer and Petrochemical Institute, Pazhoohesh Blvd., Tehran-Karaj Hwy, Tehran, 1497713115 Iran; 3 Lassonde School of Engineering, York University, 4700 Keele Street, 11 Arboretum Ln, Toronto, ON M3J 1P3 Canada

**Keywords:** Silicone acrylate, Polyhedraloligomeric silsesquioxane-acrylate, Contact lens, Hardness, Taguchi method

## Abstract

A multi-component acrylate-based copolymer system especially designed for application as ocular lenses is developed through free-radical, bulk polymerization of a system containing hydroxyethyl methacrylate, methyl methacrylate, triethylene glycol dimethacrylate, dimethyl itaconate, 3-(trimethoxysilyl) propylmethacrylate, Polyhedraloligomeric silsesquioxane-acrylate (POSS-acrylate) and AIBN as an initiator. The progress of the reaction was monitored by Fourier transform infrared spectroscopy (FTIR). The effect of increasing concentration of the components on the hardness of the synthesized lenses was measured by Shore Durometer before and after immersion in PBS solutions. Extraction test method was performed to analyze the biocompatibility of the fabricated lenses. In this research the Taguchi method was employed to achieve the optimal hardness property which plays a critical role in final application of the lens materials. The Taguchi trial for ocular lens hardness was configured in an L16 orthogonal array, by five control factors, each with four level settings. The results showed that 3-(trimethoxysilyl) propyl methacrylate decreases and 2-hydroxyethylmethacrylate increases, polyhedraloligomeric silsesquioxane with a cage-like structure, methyl methacrylate and dimethyl itaconate increase the hardness. Proliferation and growth of the cells showed that there is no toxic substance extracted from the lenses which can interfere with the cell growth.

## Introduction

In the past few years, a majority of 60% of worldwide population reported to use visual aids such as glasses or contact lenses which would rise steadily in the future (Morgan et al. 2009; Jan-Willem Bruggink et al. 2013). Therefore, a market over tens of billions of dollars per year would be predicted for this global market in ophthalmic industry (Nichols 2009).

As a brief history, ocular lenses have developed by the earliest ideas from Leonardo da Vinci to the present day. Firstly, they were made of glasses and lens materials gradually became friendlier by invention of hydrogels which were more comfortable and adjustable. High water content lenses should be thicker for more durability but increasing the thickness impairs the oxygen permeability. In the twenty-first century, silicone hydrogels allowed more than 90% of available O_2_ to reach the eyes and allowed the lenses to be thinner (Gasson and Moriss 2003). These days, the ocular lenses and color lenses are worn by many people for different purposes.

There are three types of lenses; rigid, soft and hybrid lenses due to selected materials and also final applications (Mannis et al. 2003). One of the most interesting techniques for lens preparation is in situ polymerization of multi-functional acrylic monomers, because a variety of polymerization methods and systems are possible. Free-radical bulk polymerization of vinyl monomers, characterized by auto-acceleration, has been widely investigated.

Understanding the chemical composition and effect of each component on the mechanical properties of the lenses is crucial to design the ocular lenses with the best possible performances. Lenses based on silicone acrylate monomers include the wide range of lenses; soft hydrogel lenses and rigid gas permeable hard ocular lenses (RGPL) (Mannis et al. 2003). The RGPL are made in rod-shape blank with a maximum initial diameter of 5 cm and then cut to form discs with desired thickness. This rod-shape blanks are purchased by vision centers. Ocular lenses are produced by cutting this rod-shape blanks on a lathe, polishing and finishing due to required curvatures. In other word, RGD lenses due to the presence of the blade on the surface are opaque and they need extra final finishing process. Therefore, suitable hardness of materials guarantees the quality of the cutting, finishing and polishing steps of the ocular lenses. Taguchi method is one of the best option to determine the optimal number of tests when the number of variables or factors is high (Roy 1968). Taguchi with combined orthogonal arrays was used to study the effect of five fundamental constituents of lenses, including hydroxyethyl methacrylate, methyl methacrylate, triethyleneglycol dimethacrylate, dimethyl itaconate, 3-(trimethoxysilyl) propyl methacrylate, on hardness.

## Materials and methods

### Materials

In this research hydroxyethyl methacrylate (HEMA), 3-(trimethoxysilyl) propyl methacrylate (TMSPMA), methyl methacrylate (MMA) and tetraethylene glycol dimethacrylate (TGDMA) purchased from Merck, dimethyl itaconate (DMI) and AIBN obtained from Sigma-Aldrich Chemicals, Germany, POSS-acrylate from Hybrid Plastics in USA were used. 3-[4,5-Dimethylthiazol-2-yl]-2,5-diphenyltetrazolium bromide (MTT) and DMSO were obtained from Sigma-Aldrich Chemicals, Germany.

### Methods

FTIR spectroscopy (Equinox 55, Brucker, Germany), hardness Durometer (Shore D) (Santam; Iran), vacuum oven were used to investigate the effect of composition on hardness of the synthesized lens materials.

### Experimental design

Taguchi method-based design experiments has been used to study the component concentration effects on hardness, hydrophilicity and water uptake of synthesized lens materials. The Taguchi trial for ocular lens hardness was configured in L16 orthogonal array, by five control factors, each with four level settings. Therefore, the molar concentration of initiator (AIBN) and the amount of triethylene glycol dimethacrylate (TEGDMA) as cross-linker has been considered as fixed values of 5.0 mol% and 2.8 mol%, respectively. Table 1 demonstrates the selected factors and levels and Table 2 shows 16 L used in the experimental design of Taguchi method.

**Table 1:** Selected factors and levels for Taguchi experimental design

Level	Factor				
	POSS(g)	DMI(g)	TMSPMA(g)	MMA(g)	HEMA(g)
1	0	0.15	0.2	0 24	0
2	0.05	0.20	0.3	0 28	0.05
3	0.10	0.25	0.4	0 32	0.10
4	0.15	0.30	0.5	0 36	0.15

**Table 2:** L16 orthogonal array, by five control factors, with four level settings

Factor level	HEMA	POSS	DMI	TMSPMA	MMA
1	1	1	1	1	1
2	2	2	2	2	1
3	3	3	3	3	1
4	4	4	4	4	1
5	4	3	2	1	2
6	3	4	1	2	2
7	2	1	4	3	2
8	1	2	3	4	2
9	2	4	3	1	3
10	1	3	4	2	3
11	4	2	1	3	3
12	3	1	2	4	3
13	3	2	4	1	4
14	4	1	3	2	4
15	1	4	2	3	4
16	2	3	1	4	4

### Sample preparation and polymerization

The amounts of components (based on the data in Table 1) were poured into the Petri dish at 23 °C for 30 min being homogeneously stirred using a magnetic stirrer. The weight of each batch was about 5 g. The resulting homogeneous mixture was transferred into the Teflon coated moulds and placed in the vacuum oven. Polymerization was followed in two steps; firstly they were heated up to 65 °C for 24 h and secondly, they were post-cured at 45 °C for 48 h to complete the reaction. A two-step polymerization protocol was performed where in the first step the system was below gelation (60-70 °C), and after that it was under diffusion control (40-50 °C).

### Degree of conversion measurements (DC%) using FTIR spectroscopy

FTIR analysis was used to identify functional groups well as evaluation of degree of conversion of involved monomers (Sangermano et al. 2012). For this technique, the specimens were pulverized into a fine powder and 5 mg of the ground powder were thoroughly mixed with KBr powder and then pressed using pelleting press with a load of 10 tons over 1 min to obtain a pellet. The pellets were placed into a sample holder and into the FTIR spectrophotometer.

The measurements were carried out under the following conditions: 32 scans, 4 cm^−1^ resolution, in range of 300–4000 cm^−1^ wavelength. The percentage of unreacted carbon–carbon double bonds (C=C) was determined from the ratio of the absorbance intensity of aliphatic C=C (peak at 1637 cm^−1^) against the absorbance intensity of the carbonyl group C=O (1720 cm^−1^) as a reference before and after polymerization. This experiment was carried out in triplicate. The degree of conversion was determined by subtracting the percentage of C=C from 100%, according to the formula:

The degree of conversion was calculated from Eq. (1)

1 $$ {\text {DC} {\%}} = (1- \frac {(1636/1720  {\text {cm}^{-1}})\, {\text {peak area after curing}}} {(1636/1720  {\text {cm}^{-1}})\, {\text {peak area before curing}}}) \times 100 {\%}$$.

### Hardness measurements

Hardness is the ability of materials to resist scratching and indentation which is very important in ocular lens materials. To study the surface properties, especially hardness, Shore Durometer hardness was used (ISO 868 2003). In this technique, three specimens of each group were made as discs with a diameter of 13 mm and a height of 4 mm and the hardness was calculated on four different points on the top and the bottom surfaces of each sample for 15 s. To consider the effect of environment, hardness as a model before and after exposure in PBS solution was measured.

### Biocompatibility test

The biocompatibility of medical devices is evaluated by either direct contact test or extract test methods.

In direct contact test, the samples are applied directly into the test systems such as cells or skin of animals. In extract test, the samples are immersed into the some kind of extraction media and allowed media to extract any residual chemicals which might be toxic. Then, the extraction medium is applied into the test system. In this research the extract test method was performed to evaluate the biocompatibility of the fabricated lenses. The biocompatibility tests were conducted on the extracts obtained from the samples 1, 7 and 16 as shown in Table 2. The selected samples were immersed in PBS solution at 37 °C for 28 days. The soup applied into the system test containing L929 fibroblast cells and cell behavior was studied after 24 h in accordance with ISO 10993. MTT assay was used to indicate the level of viability. The survival of fibroblast cells into the extracted was followed by 3-[4,5-dimethylthiazol-2-yl]-2,5-diphenyltetrazolium bromide. A 100 μL MTT solution with 900 μL medium was incubated with the cells in polystyrene culture plate wells (as control), and the wells with synthesized lenses at 37 °C for 3 h. Next, dimethyl sulfoxide solution was added into the dissolved formazan crystals. The absorbance values of formazan solutions were measured using an ELIZA reader at 570 nm (Bio-Tek ELx800).

## Result and discussion

**Fig. 1:**
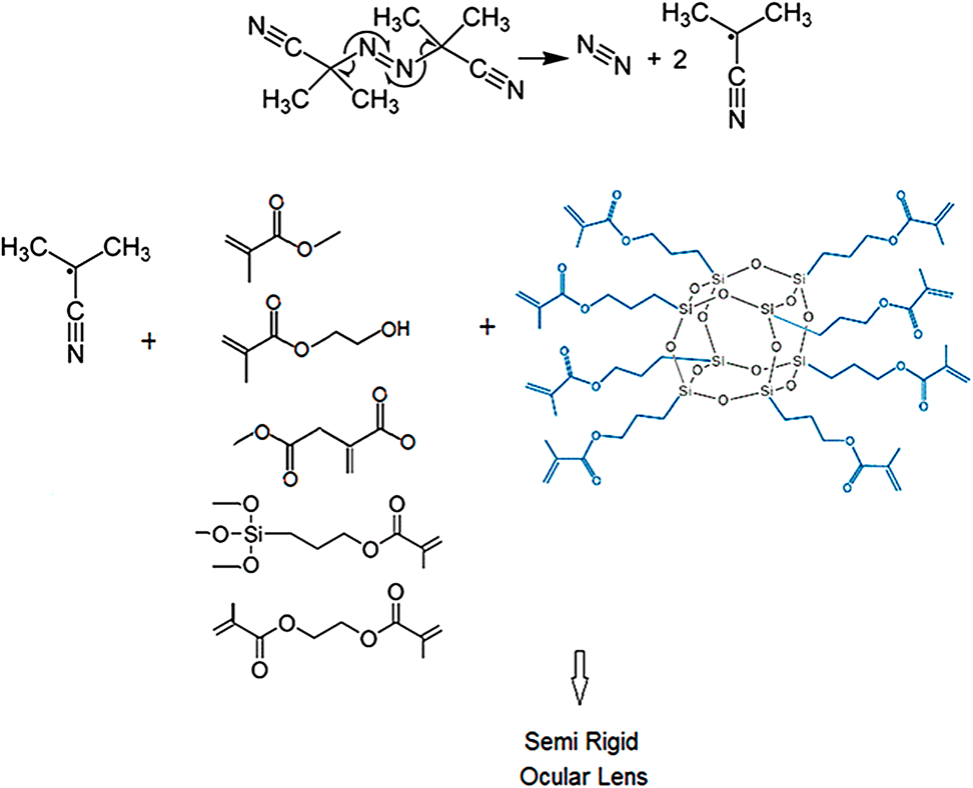
Schematic presentation of copolymerization of multi-component (meth)acrylate monomers

Free radical polymerization initiated by heat was achieved by heating the monomers at 65 °C for 24 h. As it is shown in Fig. 1 the radical attacks the double bonds of monomer, and the electron is transferred to another part. A newly formed radical attacks another monomer again and the process will be progressed and polymerization occurs. Since the materials containing silicone and oxygen have been of interest to increase the oxygen permeability (Tighe 2013, Lee et al. 2015), we introduced polyhedraloligomeric silsesquioxane (POSS)-acrylate into the polymeric backbone. Therefore, multi-functional POSS-acrylate can be polymerized in combination with HEMA, DMI, MMA, TMSPMA and TGDMA to improve the mechanical properties as well as oxygen transmission without sacrificing the other essential lens properties.

To the best of our knowledge, no studies have been published on multi-component acrylate tethered polyhedraloligomeric silsesquioxane used in ocular lenses.

**Fig. 2:**
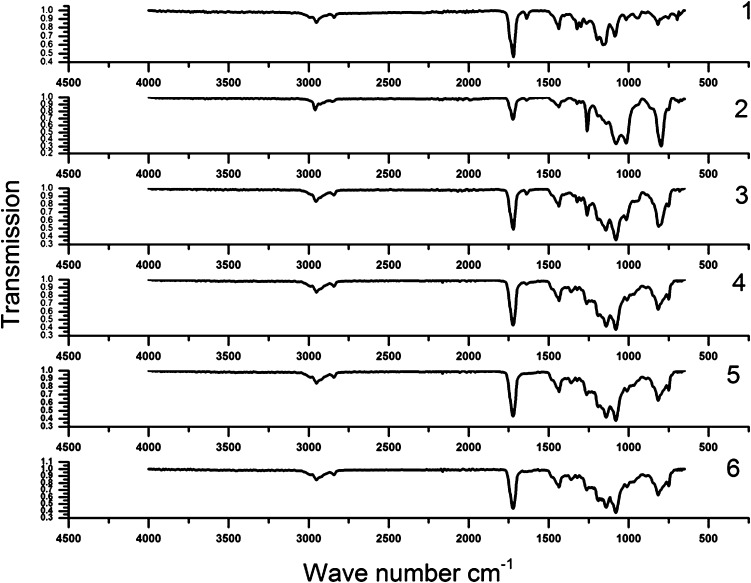
Infrared absorption spectra of samples at different reaction times; (1) 12 h (2) 24 h (3) 36 h (4) 48 h (5) 60 h and (6) 72 h

Formulation 16 of Table 2 was used to determine the suitable reaction time. The samples prepared at different temperatures were evaluated by FTIR spectroscopy to consider the reaction progress. Infrared absorption spectra obtained at different reaction times are shown in Fig. 2.

The calculated values for degree of conversion at different times are shown in Table 3.

**Table 3:** Degree of conversion in different times

Time (h)	72	60	48	36	24	12
Dc (%)	100	97	92	84	71	32

As time progresses, the degree of conversion is increased and after 48 h it reaches 92%

### Hardness study

**Fig. 3:**
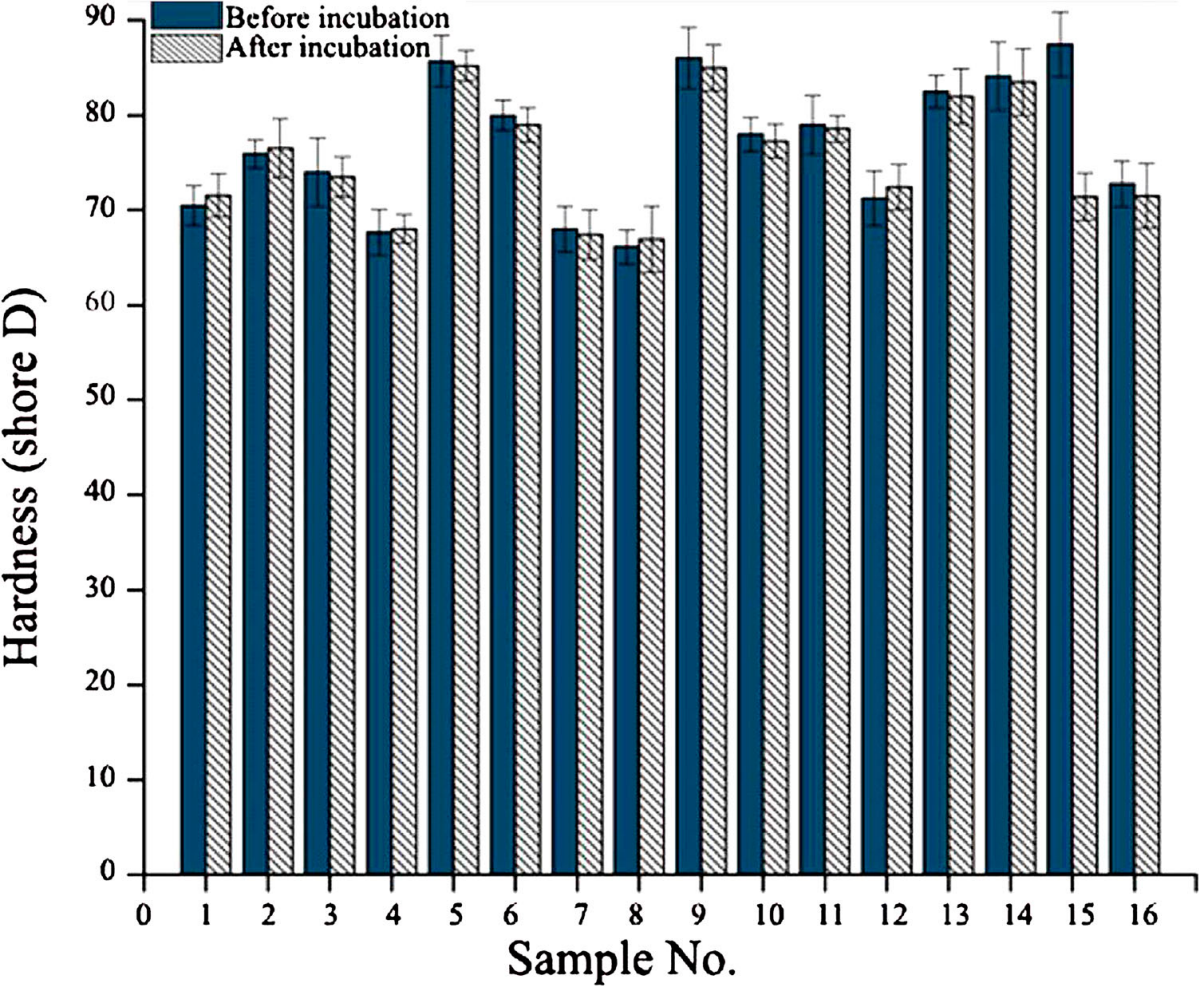
The average hardness is measured before and after immersing in PBS

Hardness is the ability of materials to resist scratching and indentation which is very important in ocular lens materials. In this study, Shore Durometer hardness was measured for 4 samples and 4 different points on each sample. Sixteen samples are available in L16 orthogonal array (Table 2), all have been tested. The average hardness was measured before and after immersing in phosphate-buffered saline (PBS). The samples were immersed in PBS for 28 days and the hardness was measured every 7 days. The average of these values is shown in Fig. 3.

Effect of monomer content on hardness was studied. The analysis of variance for the values of hardness test is presented in Table 4.

**Table 4:** Analysis of variance for the values of hardness test

Factor	(%) Participation	Pure sum of squares	Variance ratio	Variance	Sum of squares	Degree of freedom
	(*P*)	(*S*’)	(*F*)	(*V*)	(*S*)	(*F*)
MMA	27 58	2176/53	223	728	2186	3
TMSPMA	42 84	3379	346	1129	3389	3
DMI	9 69	746	79	258	774	3
POSS	13 29	1049	108	352	1058	3
Error	6 57	–	–	3/29	749	147

### Effect of trimethoxysilyl propyl methacrylate on hardness

**Fig. 4:**
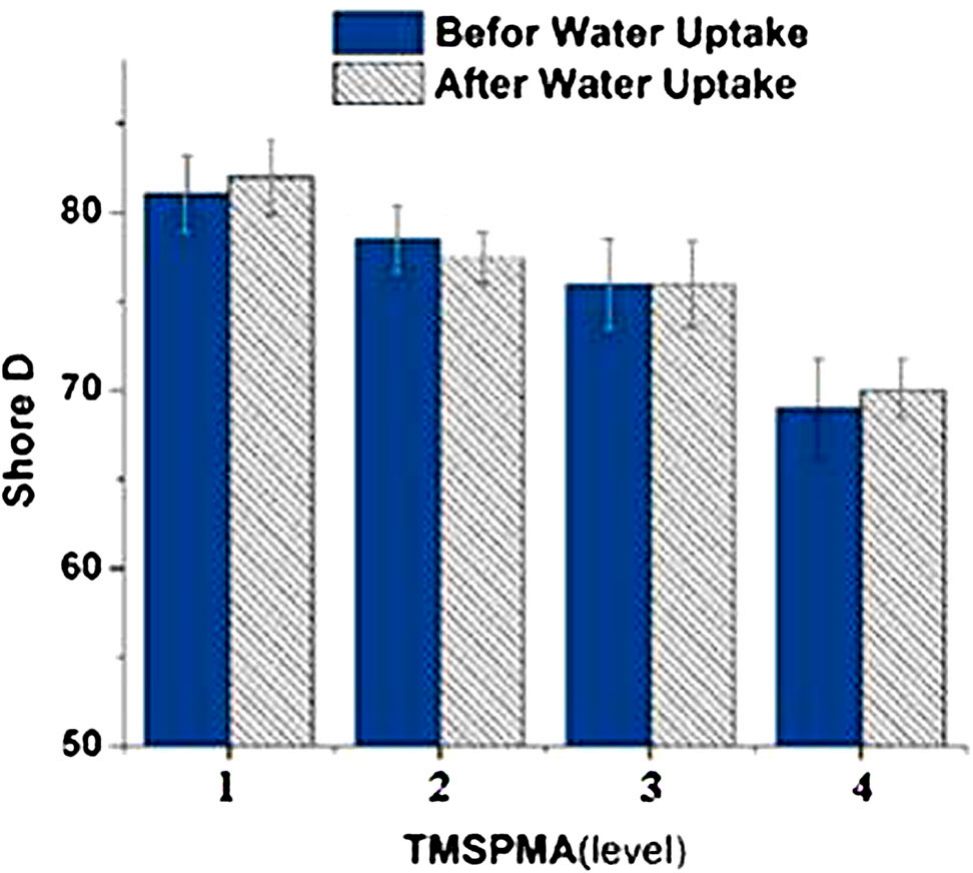
The effect of 3-methoxypropyl methacrylate alkoxysilyl on hardness hardness before and after water absorption

We expected that by entering the Si–O group into the polymer backbone, hardness would be reduced. In this study, the amount of silicone-based monomer (TMSPMA) varies from 0.2 to 0.5 g. Figure 3 shows that by increasing the amount of TMSPMA from 0.2 g in level 1 to 0.5 g in level 2, Shore D hardness has decreased by 12 units. In Fig. 4, the hardness values obtained for different amount of TMSPMA are provided after and before water absorption. The Si–O bond has 1.63 Å length and 130 degrees angle bond while C–C bond possesses 1.54 Å length and of 112 degrees bond angle (Kratky and Laggner 2002). Longer covalent bond with greater angle reduces the energy required to change the configuration of the molecules and Si–O molecules are more readily moved compared to C–C molecules. In addition to the easier movement of Si–O groups and because the lengths of branches attached to the alpha carbon and the molecular free volumes increase, there would develop easy and ready chain mobility (Mirau et al. 2001). Low energy configuration in macro-scale is comparable with reduction in hardness (Fig. 4).

### Effect of methymethacrylate on hardness

**Fig. 5:**
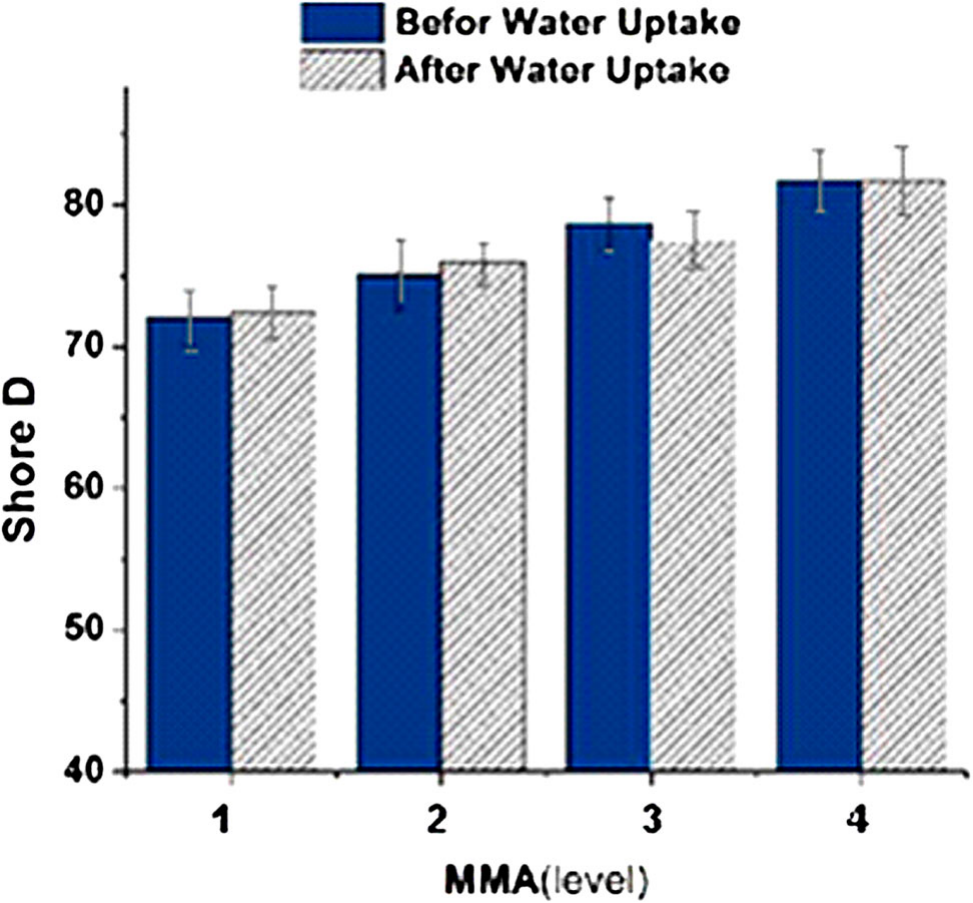
The effect of methymetacrylate on hardness before and after water absorption

Methyl groups attached to the alpha carbon of acrylic monomers leads to steric hindrance and prevents rotation around the C–C bond. In turn, this increases the required energy for movement and makes the polymer hard and brittle (Mirau et al. 2001). Figure 5 shows that the hardness increases from 73 in Shore D scale to 80; a 7 unite in Shore D.

### Effect of dimethyl itaconate on hardness

**Fig. 6:**
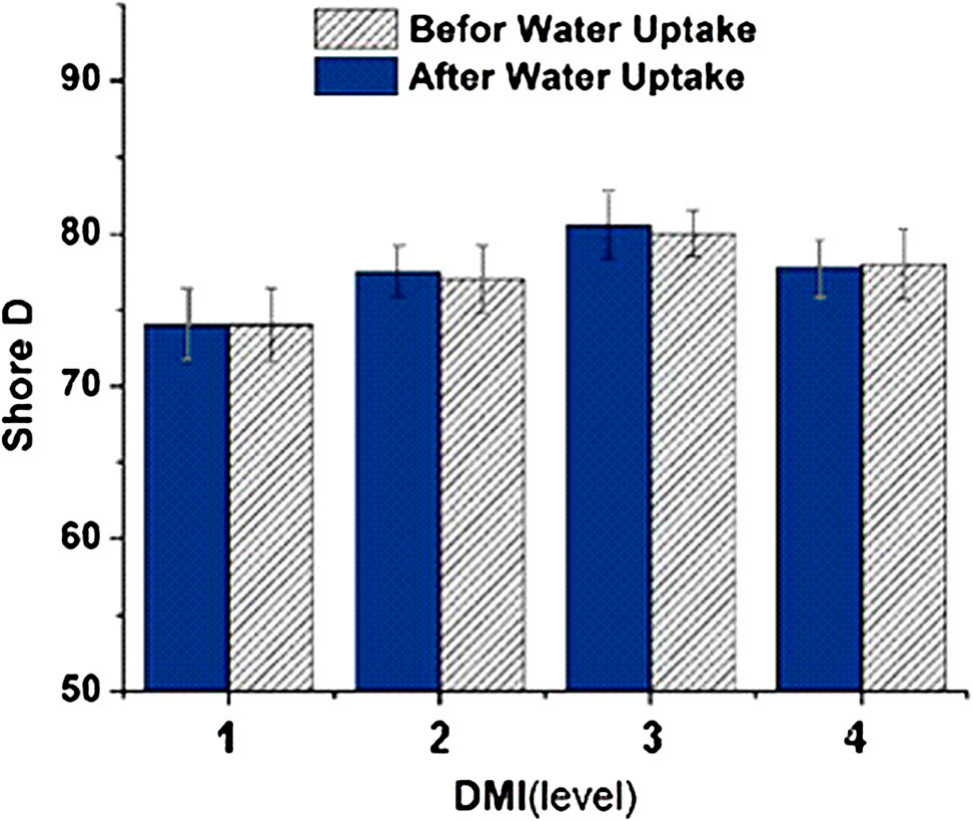
The effect of dimethyl itaconate hardness before and after water absorption

In the sample formulation, 0.15–0.3 g DMI per specimen was used. Increasing DMI increased the hardness of lens materials from 74 Shore D to 80.5 Shore D, as it is presented in Fig. 6. There are two large groups on the alpha carbon of DMI which increases the stiffness of the ocular lens materials (Saiz et al. 1988). Large groups because of steric hindrance with adjacent groups require more energy for torsion bond angle. So these groups restricted the conformational changes and also molecular mobility.

### Effect of polyhedraloligo silsesquioxane on hardness

**Fig. 7:**
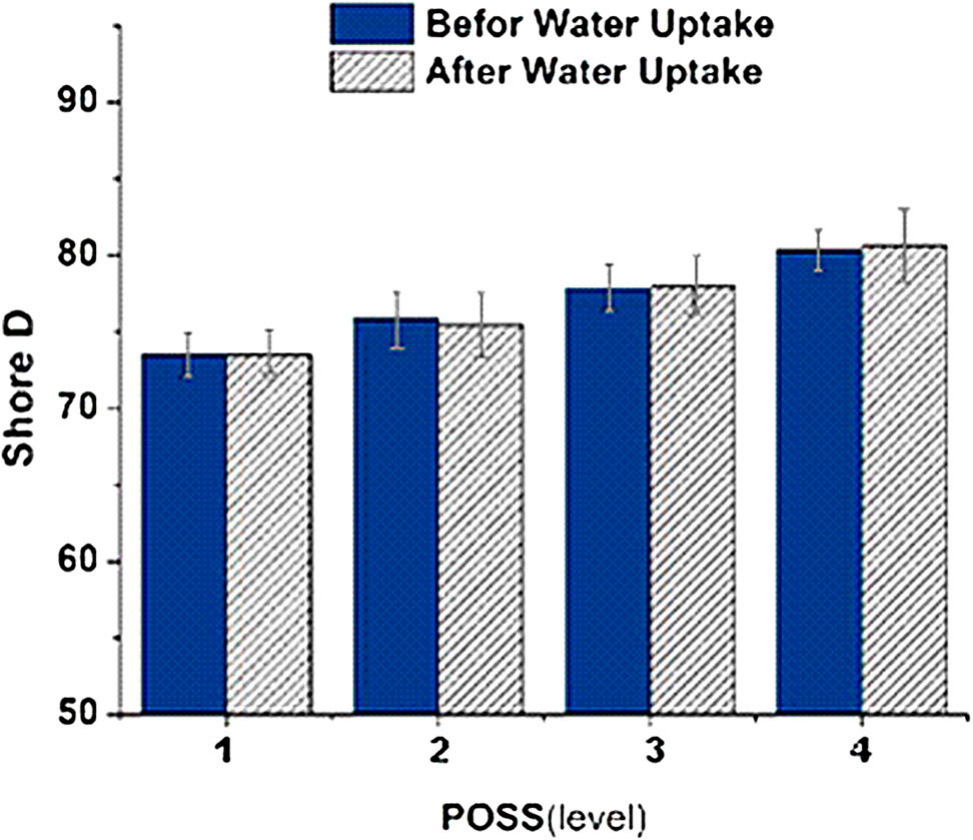
The effect of polyhedraloligo silsesquioxane on hardness before and after water absorption

Introducing POSS-acrylate increased Shore D hardness value from 73.4 to 79.6 Shore D. Existence of ten acrylate arms on the cage bridge of POSS causes the molecule to act as a crosslinking agent in the polymerization reaction. By increasing the amount of POSS, crosslinking density would increases which leads to lower segment mobility (Sperling 1992; Kopesky et al. 2004). As it is shown in Fig. 7, the reduced mobility segment has increased Shore D hardness value by 6 units.

### Biocompatibility study

**Fig. 8:**
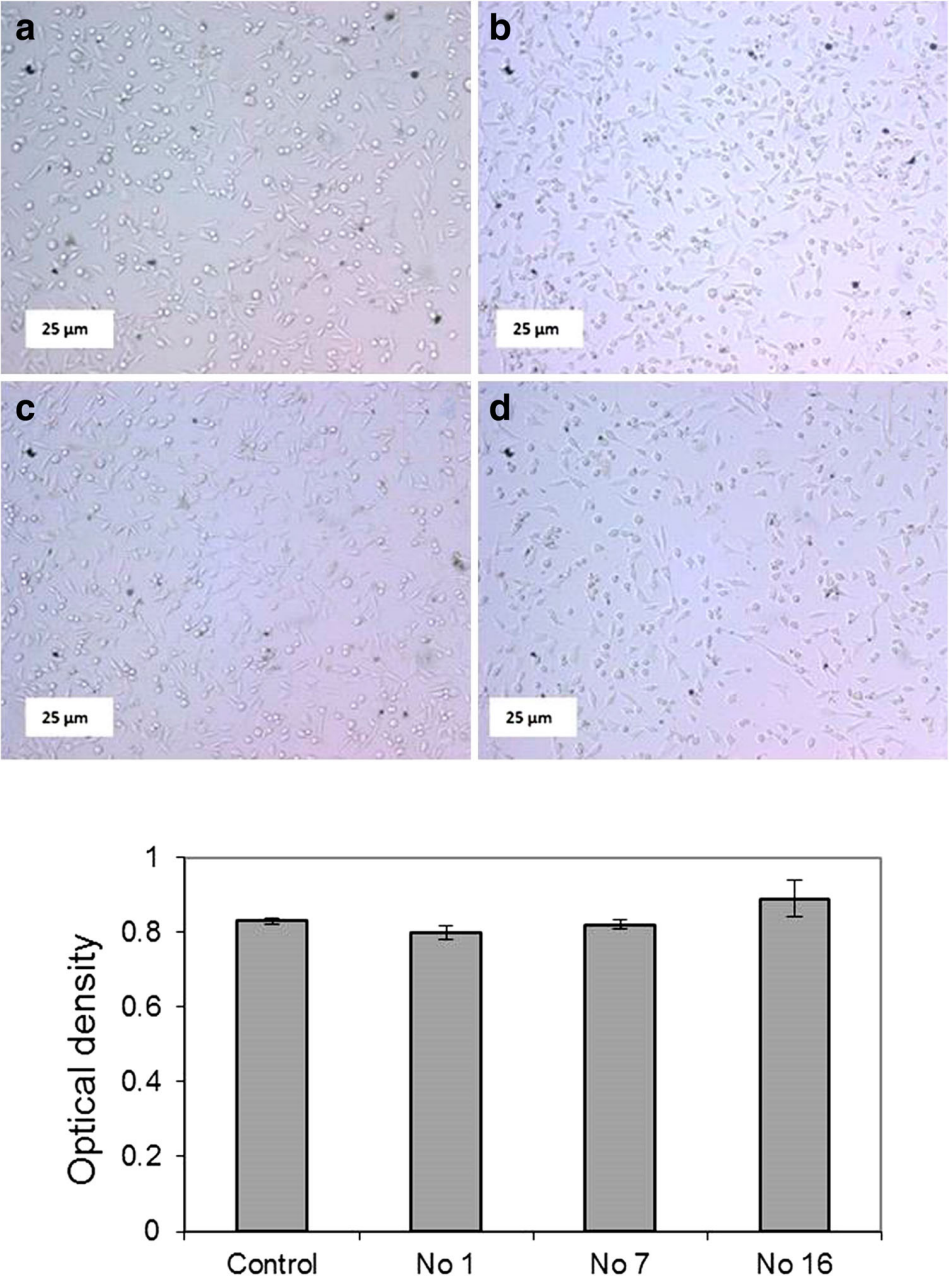
Light microscope images of L929 fibroblast cell culture in extracts from ocularlenses. (a) Control, (b) an extract prepared from a sample number of 1 in Table 2 (c) an extract prepared from a sample number of 7 in Table 2 and (d) an extract prepared from a sample number of 16 in Table 2

As it is evident in Fig. 8, the cells grow and proliferate in the extract and in comparison with the control, proliferation and growth of the cells show that there is no toxic substance in the extract media to interfere with the cell growth. The L929 cells into the extracts exhibited their typical spindle morphology and filopodia around the cells indicating the beginning of cellular expansion (Oian et al.).

Evaluated MTT results demonstrated that the level of viability confirms the results of microscopy.

## Conclusion

In this research, the experimental design Taguchi method was performed to evaluate the effect of acrylate-based components on hard ocular lenses and a multi-component silicone acrylate nanocomposite containing POSS. L16 orthogonal array was used in experimental design. The effect of increasing concentrations of the components on the scale Shore D hardness of samples before and after exposing in PBS was measured.

The tri-methoxy silyl propyl methacrylate contributes the hardness by 42% (having an impact on hardness), which is the most pronounced effect among all monomers followed by MMA. POSS, methyl methacrylate and di-methyl itaconate increases the hardness 13, 27 and 9%, respectively.

According to the results from Taguchi method, tri-methoxy silyl propyl methacrylate percentage and acrylate have great influence on controlling the hardness, while 2 hydroxymethyl methacrylate has the less impact about 3%. About 6% of total hardness observed in this study was due to errors in the test. PBS had no significant change on hardness and the difference values are negligible. Cytotoxicity test showed that the growth and proliferation of cells into the extracted media are comparable with the cells behavior in culture medium and the cells did not sense any toxic substances.
