# Correction to: Compositional design and Taguchi optimization of hardness properties in silicone-based ocular lenses

**DOI:** 10.1007/s40204-018-0080-7

**Published:** 2018-01-23

**Authors:** Mohammad Hanifeh, Mojgan Zandi, Parvin Shokrollahi, Mohammad Atai, Ebrahim Gafar-Zadeh, Fahimeh Askari

**Affiliations:** 1 Biomaterials Department, Iran Polymer and Petrochemical Institute, Pazhoohesh Blvd., Tehran-Karaj Hwy, Tehran, 1497713115 Iran; 2 Science Department, Iran Polymer and Petrochemical Institute, Pazhoohesh Blvd., Tehran-Karaj Hwy, Tehran, 1497713115 Iran; 3 Lassonde School of Engineering, York University, 4700 Keele Street, 11 Arboretum Ln, Toronto, ON M3J 1P3 Canada

## Correction to: Prog Biomater (2017) 6:67–74 10.1007/s40204-017-0065-y

The original version of this article unfortunately contained a mistake: The spelling of the Ebrahim Gafar-Zadehs’ name was incorrect. The corrected name is given above.

